# Transforming Traditional Flatbread (Bazlama) into a Functional Food with Very High Resistant Starch and Low Glycemic Impact

**DOI:** 10.3390/foods15101752

**Published:** 2026-05-15

**Authors:** Cagla Ozer, Halide Yildirim, Ece Surek, Kubra Ozkan, Osman Sagdic, Samuela Palombieri, Francesco Sestili, Hamit Koksel

**Affiliations:** 1 Department of Gastronomy and Culinary Arts, Faculty of Fine Arts, Design and Architecture, Istinye University, 34396 Istanbul, Türkiye; cozer@istinye.edu.tr (C.O.); halide.yildirim@istinye.edu.tr (H.Y.); kubraozkan1907@gmail.com (K.O.); 2 Institute of Nanotechnology and Biotechnology, Istanbul University-Cerrahpasa, 34500 Istanbul, Türkiye; ece.surek@iuc.edu.tr; 3 Department of Food Engineering, Faculty of Chemical and Metallurgical Engineering, Davutpasa Campus, Yildiz Technical University, 34349 Istanbul, Türkiye; osagdic@yildiz.edu.tr; 4 Department of Agriculture and Forest Sciences (DAFNE), University of Tuscia, 01100 Viterbo, Italy; palombieri@unitus.it

**Keywords:** flatbread (bazlama), high amylose, resistant starch texture, glycemic index, antioxidant capacity

## Abstract

This study investigated the reformulation of traditional Anatolian flatbread (bazlama), a staple food of the Mediterranean diet, into a functional product with enhanced nutritional quality. High-amylose refined (white) flour obtained from high-amylose Svevo (Svevo-HA) wheat and resistant starch produced via repeated autoclaving–cooling cycles were incorporated to increase resistant starch content and antioxidant capacity, reduce the predicted glycemic response, and evaluate the resulting changes in textural attributes. Six bazlama formulations were produced using white flours of normal Svevo, Svevo-HA, and recombined Svevo-HA flour containing resistant starch and gluten, with and without vital gluten supplementation. Color, texture profile, phenolic content, antioxidant capacity (DPPH, ABTS, FRAP), resistant starch content, and in vitro glycemic index (GI) were evaluated. Bazlama samples enriched with resistant starch exhibited significantly higher total antioxidant activity (113.7–174.7 mg Trolox equivalent/100 g dw) and resistant starch (9.1–10.3%) levels, along with reduced GI values (53.8–54 < 55), classifying them as low-GI foods. The results demonstrate that incorporating high-amylose wheat–derived resistant starch can successfully convert bazlama into a functional flatbread with improved health-promoting properties.

## 1. Introduction

Wheat and wheat-based foods are fundamental components of traditional diets worldwide and have played a central role in human nutrition since ancient times [1]. Due to its high consumption, technological versatility, and favorable sensory attributes, wheat has become the most widely cultivated food crop and the most accepted cereal food ingredient around the world [2]. Accordingly, wheat represents a highly promising platform for improving human health through targeted modifications of its composition and its incorporation into widely consumed food products. Even small improvements in the nutritional profile of wheat can have a substantial impact on dietary quality across entire populations, contributing to increased dietary intake of health-promoting components such as resistant starch and thereby contributing to a reduced glycemic response. In this regard, wheat-based products enriched with higher amylose content offer significant potential as a sustainable and effective dietary source of resistant starch for global public health [3]. Resistant starch (RS) is the fraction of starch that escapes digestion in the small intestine and undergoes fermentation in the colon, producing short-chain fatty acids (SCFAs) that contribute to gut health and influence systemic metabolism [4]. High-amylose wheat has emerged as an effective raw material for increasing RS content in cereal-based foods. The elevated amylose fraction promotes the formation of compact crystalline structures and amylose–lipid complexes that limit enzymatic accessibility, thereby reducing starch digestibility and postprandial glycemic response. As a result, high-amylose wheat-based products have gained increasing attention as candidates for low-glycemic staple foods [5,6,7].

Bread, a staple food consumed worldwide, has frequently been used as a model system for nutritional enhancement [8]. Flatbreads, among the most ancient forms of bread, are widely consumed across Mediterranean, Middle Eastern, and Asian cuisines. They exist in wide varieties with distinct quality characteristics, yet all share the same basic ingredients: flour, salt, water, and yeast [8]. Bazlama is a traditional Anatolian flatbread described as a single-layered, circular, leavened bread with a creamy yellow color, a diameter ranging from 10 to 20 cm, an even thickness of about 3 cm, and a soft texture [9]. Although some studies have examined the enrichment of bazlama with functional ingredients [9,10], the use of high-amylose wheat-derived RS remains insufficiently studied. Most studies on RS and functional bakery products have primarily focused on loaf-type breads or other baked goods, while traditional products such as bazlama have been largely overlooked [7,11,12].

The present study aims to address these knowledge gaps by investigating the potential application of high-amylose wheat and RS as functional ingredients in flatbread production. The primary objective was to evaluate the nutritional properties and quality characteristics of bazlama samples enriched with high-amylose wheat flour, RS prepared through repeated autoclaving–cooling cycles from Svevo High-amylose (HA) starch, and vital gluten. Specifically, bazlama samples prepared from white flours of normal Svevo, flour of Svevo-HA (SHA), and recombined Svevo-HA flour (rSHA), with and without vital gluten supplementation, were compared in terms of color, texture, RS content, phenolic composition, antioxidant capacity, and in vitro glycemic index. The overall objective was to develop a traditional flatbread with enhanced health-promoting properties while maintaining acceptable quality characteristics. It was hypothesized that RS-enriched recombined Svevo-HA flour would increase resistant starch content and reduce in vitro glycemic index, while vital gluten would partially improve texture.

## 2. Materials and Methods

### 2.1. Materials

Svevo and Svevo-HA durum wheats were cultivated at the experimental farm “Nello Lupori” of the University of Tuscia (Viterbo, Italy) during the 2023–2024 growing season and used as raw materials for bazlama production. Wheat samples were milled into flour according to the procedure of the AACC-approved method 26–50 [13] using a Buhler MLU 202 Pneumatic Laboratory Mill (Uzwil, Sweden). Commercial vital gluten was supplied by Tereos Starch & Sweeteners (Belgium N.V., Genk, Belgium).

### 2.2. Resistant Starch Formation from Svevo-HA Flour

Starch and gluten were separated from Svevo-HA flour. The separation process consisted of dough-making and manual washing to separate gluten. Dough was prepared using 500 g of flour at 60% water absorption and mixed in a mixer (KitchenAid Artisan Mini 3.5 Quart Stand Mixer, KSM3316X, Benton Harbor, MI, USA) until dough development (6 min). The dough was rested for 10 min, divided into 50 g portions and manually washed under tap water running in drops to remove starch and other soluble constituents. The slurry containing starch and soluble material was collected, transferred into trays at a height of 2–3 mm and then dried at 40 °C in an oven with the exhaust vent kept open (Skyline Premium, Electrolux Professional, Stockholm, Sweden). The dried starch-rich fraction was collected for subsequent processing steps. The gluten was frozen at −55 °C and freeze-dried (Teknosem, Istanbul, Türkiye). Dry starch and gluten were ground in a grinder (Fakir Aromatic, Vaihingen an der Enz, Germany).

For RS formation, the method by Koksel et al. [14] was used with slight modifications. Briefly, 400 g of Svevo-HA starch was dispersed in boiling water at a 1:10 (*w*/*v*) ratio and heat-treated by boiling for 30 min, followed by autoclaving at 121 °C for 60 min. The autoclaved starch was then subjected to enzymatic debranching using pullulanase (Promozyme, 400 PUN/mL; 2000 U/kg starch) and incubated at 60 °C for 66 h. After enzymatic treatment, the dispersion underwent an autoclaving step at 121 °C for 30 min and was subsequently cooled and stored at 4 °C for 24 h. This autoclaving–cooling cycle was repeated four times to enhance RS formation. Finally, the modified starch was dried at 40 °C and milled to obtain the RS in powder form.

### 2.3. Bazlama Production

The bazlama samples were produced as described by Beyaz et al. [9]. The bazlama formulation consisted of 100 g of flour, dry yeast (2%), salt (1.5%), and sugar (1%), combined with water (65%) at 30 °C. All ingredients were mixed using a stand mixer to form the dough. The dough was placed in an Electrolux Skyline Chill (Electrolux, Stockholm, Sweden) at 30 °C with 85% relative humidity for 1 h. After fermentation, the dough was divided into two equal portions and rounded manually. These pieces were covered with stretch film and allowed to rest at room temperature (22–24 °C) for 6 min. Subsequently, the dough was rolled out to a uniform thickness of 7 mm using a rolling pin. The prepared dough sheets were baked in a preheated pizza electric skillet (Goldmaster, Istanbul, Türkiye) at 200 °C for 12 min. To ensure uniform heat distribution and equal baking on both sides, the bazlama samples were flipped after cooking for 6 min, and the other side was baked for the same time period. Once the samples were baked, they were cooled at room temperature, sealed in plastic bags, and stored at room temperature for subsequent analyses.

Two different bazlama samples were produced using flour from Svevo and Svevo-HA flour. Two other bazlama samples were produced by adding vital gluten (based on wheat flour weight) to Svevo flour (S) and Svevo-HA flour. The flour of Svevo-HA (SHA) was separated into gluten and starch, and the starch part was processed to enrich its RS content, as explained above. Then, the RS-enriched starch obtained from the starch part of Svevo-HA and its gluten were recombined in their original ratios to form a new flour comprising Svevo-HA gluten (12%) and RS from SHA (88%). The recombined SHA is called rSHA throughout the manuscript. The amount of wet gluten and dry gluten was determined according to AACC 38–12 [13]. Two other bazlama samples were also produced from rSHA (one with and one without gluten). To summarize, bazlama samples were produced in six different formulations: three without vital gluten addition (S, SHA, and rSHA) and three with vital gluten (2%) addition to these three flours.

### 2.4. Analyses of Bazlama Samples

Crust and crumb color of bazlama samples were evaluated using a colorimeter (Konica Minolta CR-400, Osaka, Japan). Color parameters were reported in the CIE L*a*b* system, where L* represents lightness (0 = black, 100 = white), a* indicates red–green values (positive values = red, negative values = green), and b* corresponds to yellow–blue values (positive values = yellow, negative values = blue). Prior to measurements, the instrument was standardized using a white calibration plate.

Texture profile analysis (TPA) was performed with a Texture Analyzer (TA.XT2 Plus, Stable Micro Systems Ltd., Surrey, UK) following the procedure described by Koksel et al. [10].

The bazlama samples were divided into roughly four equal slices. For TPA, pre-test speed, post-test speed, and test speed were each set at 1.7 mm/s, and the compression was 30% of the height of the bazlama sample. An interval of 5 s between two compression cycles and a trigger force of 5 g were selected. A P/36 cylindrical probe (diameter: 36 mm; Stable Micro Systems, Surrey, UK) was used for TPA. TPA curves provided the parameters of hardness, springiness, cohesiveness, gumminess, chewiness, and resilience.

The RS content of bazlama samples was quantified according to AACC Method 32–40.01 [13] using a commercial assay kit (Megazyme International, Wicklow, Ireland). Sample digestion was conducted based on the method of Kahraman et al. [15]. Glucose levels were determined using a glucose oxidase–peroxidase reagent kit (Megazyme International, Wicklow, Ireland). From these data, the in vitro starch hydrolysis index (HI) and the estimated glycemic index (GI) were calculated. The in vitro GI was determined by using the following equation:

GI = 39.71 + (0.549 × HI)

Free and bound phenolic compounds were extracted as described by Koksel et al. [10]. Their concentrations were measured using the Folin–Ciocalteu assay [16], and results were expressed as mg of gallic acid equivalents (GAE) per 100 g of sample on a dry weight basis. Total phenolic content (TPC) was obtained by summing free and bound fractions.

Antioxidant capacity was assessed using ABTS radical scavenging activity, DPPH free radical scavenging assay, and ferric reducing antioxidant power (FRAP), following the methods of Re et al. [17], Singh et al. [18], and Benzie and Strain [19], respectively. Results were expressed as milligrams of Trolox equivalents (TE) per 100 g dry weight.

### 2.5. Statistical Analysis

All analyses were conducted in triplicate, and the results are expressed as mean values accompanied by their standard deviations. Statistical differences among samples were assessed using one-way analysis of variance (ANOVA), followed by Tukey’s multiple comparison test. The analyses were performed with SPSS Statistics software (IBM, version 20). A significance level of *p* < 0.05 was applied to determine statistically meaningful differences.

## 3. Results and Discussion

### 3.1. Color Properties of Bazlama Samples

Color attributes are key quality indicators influencing consumer perception and acceptance of flatbread products [20]. The crumb and crust color parameters (L*, a*, and b*) of bazlama samples are presented in Table 1. The highest lightness (L*) values for both crumb and crust (75.81 and 74.15) were observed in bazlama prepared from Svevo flour, indicating a brighter product. Vital gluten supplementation did not significantly affect color parameters in the samples prepared from S or SHA. On the other hand, the L* values of the crumb and crust of bazlama samples prepared using rSHA (with and without vital gluten) were significantly (*p* < 0.05) lower compared to all other samples, indicating an increase in grayish color. L*, a*, and b* values of bazlama samples were found to range from 48.19 to 75.81, from −2.56 to 3.92, and from 22.36 to 27.36, respectively, for crumb; and from 44.17 to 74.15, from −2.12 to 8.76, and from 27.84 to 30.94, respectively, for crust. These values are consistent with those reported for control flatbread samples in previous studies [8,20].

**Figure 1:**
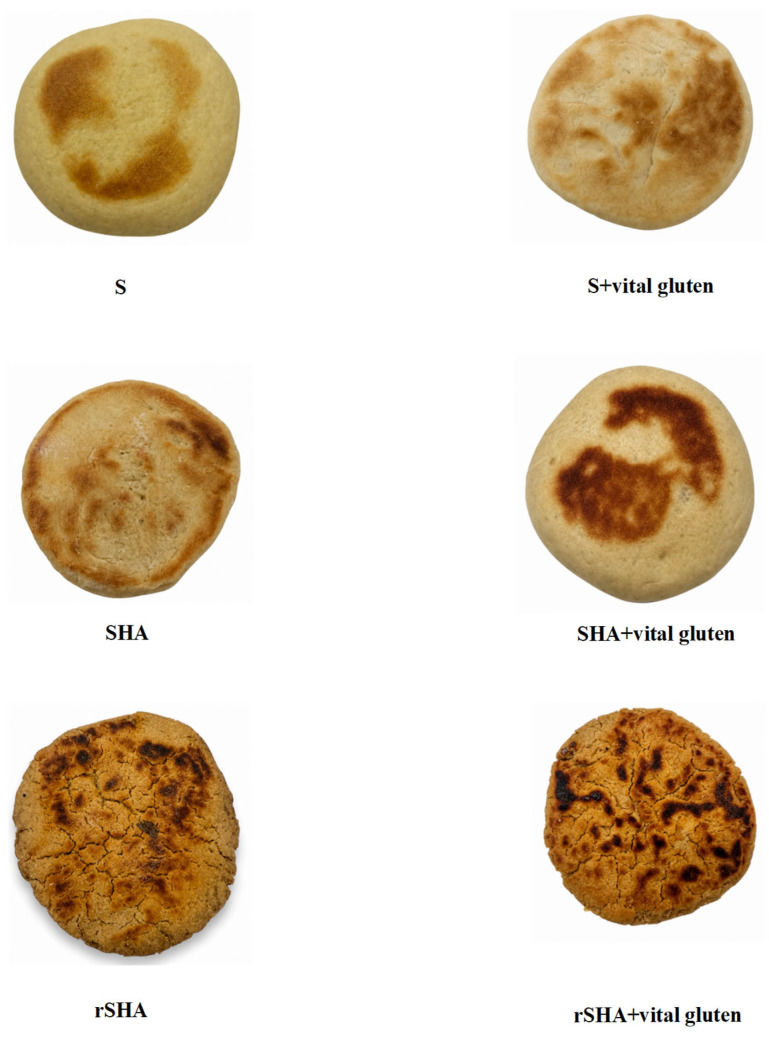
Images of bazlama samples. S: Svevo flour, SHA: Svevo-HA flour, rSHA: recombined flour from heat-treated Svevo-HA starch and Svevo-HA gluten.

The relatively higher b* values observed in this study, compared with some literature reports (16.17, [21]; 17.08–19.64, [22]), may be related to the use of durum wheat flour, which is known to impart enhanced yellowness due to its carotenoid content. In a study by Ertaş [11], the L* color value of a control bazlama sample containing no bran or whole wheat flour (and including 1.4% vital gluten) was reported as 75.21, which is higher than the L* values determined for the vital-gluten-containing samples in the present study. Consistent with our results, Andrzej et al. [23] reported that the control group exhibited the highest crumb brightness (L* = 70.65), whereas adding barley flour to bread formulations led to a reduction in the L* value (66.07). In another study, Beyaz et al. [9] reported that the crumb and crust L* values decreased as the barley flour supplementation level increased in the bazlama samples. The bazlama samples produced from rSHA exhibited significantly higher crumb and crust a* color values than those prepared using only S or SHA (*p* < 0.05). The bazlama samples prepared using rSHA and rSHA+vital gluten showed lower crumb b* yellowness values than the other samples (Table 1). However, no significant differences were observed among the crust b* color values of the bazlama samples (*p* > 0.05). The images of bazlama samples are shown in Figure 1.

### 3.2. Textural Properties of the Bazlama Samples

Texture profile analysis results are summarized in Table 2. Crumb hardness is a critical parameter influencing bread quality and consumer preference. Bazlama samples prepared from rSHA and rSHA+vital gluten exhibited significantly higher hardness values (49.18 N and 43.86 N, respectively) than those produced from S or SHA flours (*p* < 0.05), likely due to the reduced availability of digestible starch and modifications in the starch–protein network caused by RS formation.

Vital gluten supplementation significantly reduced hardness across all flour types, indicating partial compensation for the weakened gluten structure associated with high RS content. However, even with gluten addition, rSHA samples remained firmer than the control samples, suggesting that further formulation or processing optimization may be required to improve softness. Similar to this result, in a study using legume flour as a substitute for wheat flour, Cacak-Pietrzak et al. [24] observed an increase in the crumb hardness of bread. Hardness is regarded as the primary textural attribute of bread, and a softer crumb is generally considered more desirable in terms of consumer preference. Therefore, it can be concluded that the utilization of a gluten and RS mixture as a flour source in bazlama production may not be enough to obtain the desired texture, and textural properties needed to be further developed by changing the supplementation levels of those ingredients or using other techniques to improve the texture of the flatbread made from rSHA flour.

Cohesiveness, springiness, and resilience are additional key textural attributes that are largely determined by gluten quality [25]. Low cohesiveness suggests that the bread crumb is fragile and easily disintegrates, which can adversely affect consumer acceptance. In this study, the highest cohesiveness value (0.91) was found in the bazlama sample prepared using S and vital gluten. However, no significant difference was detected in the cohesiveness values of other samples, except for rSHA. The effect of vital gluten supplementation was determined to be significant for only the cohesiveness values of the rSHA bazlama samples (*p* < 0.05). The addition of vital gluten was able to increase the cohesiveness of the rSHA bazlama sample. The difference between springiness values of bazlama samples produced from S or SHA was not significant (*p* > 0.05). Moreover, the addition of vital gluten did not lead to any change in springiness or resilience values for any of the samples. The significant increase in hardness following gluten supplementation can be attributed to the increased density of the protein matrix. However, springiness and resilience characteristics, which are related to elasticity, remained consistent since gluten enrichment can enhance the mechanical resistance of the bread without changing the recovery dynamics of the protein-starch matrix after compression. Li et al. [7] focused on the role of high amylose content in the textural quality of bread in their research. Bread prepared with high-amylose wheat flour exhibited higher hardness and lower resilience values compared to bread with wild-type wheat flour. The greater hardness of high-amylose bread was attributed to its dense and compact structure, and the resilience of the bread was negatively related to hardness. The springiness (0.83–0.85) and resilience values (0.43–0.47) of rSHA and rSHA+vital gluten bazlama samples were significantly lower than those of other samples (0.97–0.98; 0.55–0.62). A significant difference was found between resilience values of vital gluten-supplemented bazlama samples prepared from S (0.62) and SHA (0.56) flours. Since springiness and resilience are interrelated properties, a decline in their values reflects a loss of elasticity in the bread crumb. Significantly higher gumminess (24.72–25.22) and chewiness values (21.99–22.48) were determined for bazlama samples produced using SHA and rSHA (*p* < 0.05). The vital gluten-supplemented bazlama samples prepared from S possessed the lowest gumminess and chewiness values, 14.71 and 14.30, respectively. The supplementation of vital gluten was found to be significant for only the chewiness of the sample produced from S; however, it did not create any significant difference in gumminess and chewiness values of other samples.

### 3.3. Resistant Starch Contents and Estimated HI and In Vitro GI Values of the Bazlama Samples

RS, HI, and in vitro GI values of the bazlama samples are shown in Table 3. The RS content of the RS powder we produced was determined to be 12.06 ± 0.23%. The RS contents were significantly (*p* < 0.05) higher in bazlama samples prepared from SHA (2.08 and 2.38%) than in those prepared from S (0.95 and 0.96%). As expected, the use of SHA in bazlama production resulted in significant increases in RS content compared to the control group (S). Much higher and statistically significant (*p* < 0.05) increases in RS values were observed when bazlama samples were produced using rSHA, which was obtained by recombining starch prepared through repeated autoclaving–cooling cycles from Svevo-HA starch with its freeze-dried gluten. The RS content of the rSHA bazlama sample reached 10.30%. The same flour was also used in bazlama production with the addition of vital gluten to improve textural properties as well as crust and crumb color. Although the RS content of the rSHA + vital gluten bazlama sample (9.12%) was slightly lower than that of the rSHA sample, it remained significantly higher than that of the control group. Due to higher RS content, in vitro GI values of the bazlama samples produced using rSHA (with and without vital gluten addition) were significantly lower (53.78 and 54.00) than those of bazlama samples produced from S (74.06 and 74.80) and SHA (73.32 and 73.84), both with and without vital gluten addition (*p* < 0.05). In contrast, the addition of vital gluten did not significantly affect the GI values of bazlama samples. According to the classification proposed by Kumar et al. [26], foods with GI values above 70 are classified as high-GI, those below 55 as low-GI, and those between 56 and 69 as medium-GI foods. Based on this classification, the results indicate that it was possible to convert bazlama from a high-GI product to a low-GI product by replacing its native starch with RS-enriched starch prepared through repeated autoclaving–cooling cycles from Svevo-HA starch. The GI values of rSHA and rSHA+vital gluten flatbread samples were among the lowest ones obtained for white flour breads in the related literature.

Bakery products such as bread, cakes, and biscuits are generally categorized as high-GI foods due to their high content of rapidly digestible starch and low dietary fiber levels. In recent years, considerable research effort has focused on improving the nutritional quality of bakery products through the incorporation of functional ingredients. The GI values of rSHA and rSHA+vital gluten samples treated with pullulanase and autoclaving–cooling cycles were significantly lower than those of the other bazlama samples (*p* < 0.05), classifying them as low-GI foods. This reduction can be attributed to the increased formation of resistant starch, particularly RS3, promoted by enzymatic debranching and subsequent retrogradation during repeated autoclaving–cooling cycles. The resulting ordered structures are less susceptible to enzymatic hydrolysis, thereby slowing glucose release. In contrast, SHA flour alone remains more accessible to digestive enzymes, leading to a comparatively higher GI level [14]. In this context, Beyaz et al. [9] demonstrated that the incorporation of barley or lentil flour into bazlama formulations significantly reduced in vitro GI values (*p* < 0.05). Another important parameter associated with starch digestibility is the HI, which is calculated as the ratio of the area under the hydrolysis curve of a test sample to that of a reference sample (white bread) over the same time period [27]. The HI of rSHA and rSHA+vital gluten bazlama samples was significantly lower than that of the other bazlama samples, further confirming their reduced starch digestibility.

### 3.4. Phenolic Contents and Antioxidant Capacities of the Bazlama Samples

Phenolic compounds, found in free and bound forms in cereals and legumes, act as potent antioxidants by scavenging reactive radicals, inhibiting lipid peroxidation, and chelating iron [28]. The phenolic content and antioxidant capacity values (free, bound, and total) of the bazlama samples are shown in Table 4. The concentration of bound phenolics (300.29–326.67 mg GAE/100 g dry weight (dw)) was significantly higher than that of free phenolics (211.93–240.44 mg GAE/100 g dw) for all samples (*p* < 0.05). Vital gluten supplementation led to significant increases in free phenolic content of bazlama samples, whereas the changes in bound phenolic content were not statistically significant (*p* > 0.05). The total phenolic contents of bazlama samples produced using S and SHA ranged from 512.22 to 546.60 mg GAE/100 g dw. Significantly higher (*p* < 0.05) total phenolic contents were observed when bazlama samples were produced using rSHA. The total phenolic content of the rSHA bazlama sample was 552.23 mg GAE/100 g dw. This effect is likely attributed to the enhanced extractability of phenolic compounds following autoclaving–cooling cycles. Furthermore, the combined application of enzymatic hydrolysis and autoclaving–cooling treatment may further influence the release and availability of these phenolic compounds [14]. The same flour was also used in bazlama production with the addition of vital gluten to investigate changes in phenolic contents and antioxidant activity, resulting in a further significant increase in phenolic content (567.40 mg GAE/100 g dw). The total phenolic content values found in this study were higher than those reported for bazlama samples in previous studies (53.00, [22]; 114, [29]; 112 mg GAE/100 g, [21]). Given the complex nature of cereals and cereal-based products, multiple methods are required to adequately evaluate their antioxidant capacities. Accordingly, three methods (ABTS, DPPH, and FRAP) were employed in the present study. Consistent with the trend observed in phenolic content values, rSHA (with and without vital gluten) in bazlama formulations resulted in a significant increase in the antioxidant capacities for both free and bound fractions across all methods compared to the other bazlama samples. The highest total antioxidant values were determined for the rSHA+vital gluten bazlama sample with values of 174.74, 165.78, and 140.62 mg TE/100 g dw as determined by the FRAP, ABTS, and DPPH methods, respectively. The addition of vital gluten to the bazlama samples resulted in a statistically significant increase in antioxidant capacity as determined by the ABTS and DPPH assays. An increase was also observed in the FRAP values; however, this was not statistically significant. For comparison, total antioxidant activity of a protein-rich flatbread prepared using millet, pulses, and oilseed flours was reported as 120.64 mg TE/100 g by the DPPH method [20], while values of 94.86 mg TE/100 g (DPPH) and 54.05 mg TE/100 g (ABTS) were reported for a flatbread prepared using 100% wheat flour [29]. The DPPH-based antioxidant capacity reported in these studies was within a similar range to that observed in the present study (60–140 mg TE/100 g dw), whereas the ABTS-based values were comparatively lower than those obtained here (76–165 mg TE/100 g dw). The results showed that Svevo wheat, Svevo-HA wheat, and rSHA had promising antioxidant potential to be used in flatbread formulation.

## 4. Conclusions

This study demonstrated the feasibility of transforming traditional Anatolian flatbread (bazlama) into a functional food through the incorporation of high-amylose wheat–derived RS. The use of SHA and rSHA enriched with RS significantly enhanced the nutritional quality of bazlama by increasing RS and phenolic contents, improving antioxidant capacity, and markedly reducing the in vitro GI. Bazlama samples produced from rSHA exhibited low-GI values (<55), indicating the potential for the development of products with a lower predicted glycemic response; however, this should be verified in human studies. Although RS incorporation led to increased crumb firmness and reduced elasticity, partial improvement was achieved through vital gluten supplementation, highlighting the importance of formulation optimization to balance nutritional and textural quality. Overall, the findings confirm that high-amylose wheat and processing-induced RS formation can be effectively applied to produce traditional flatbread with enhanced nutritional properties, although further optimization is needed to improve texture and appearance. Future studies should also focus on in vivo testing of glycemic control, sensory evaluation and consumer acceptance to support large-scale applications. The development of such functional flatbreads from white flours aligns with current dietary recommendations and offers promising opportunities for improving the health profile of staple foods within the Mediterranean diet.

## Figures and Tables

**Table 1:** Color values of the bazlama samples.

	Crumb Color			Crust Color		
Flours Used in Bazlama	L*	a*	b*	L*	a*	b*
S	75.81 ± 0.43 ^a^	−2.35 ± 0.15 ^c^	27.36 ± 0.64 ^a^	74.15 ± 1.52 ^a^	−1.41 ± 0.43 ^b^	30.08 ± 1.05 ^a^
S+vital gluten	71.31 ± 0.22 ^a^	−2.56 ± 0.11 ^c^	25.76 ± 1.79 ^ab^	72.97 ± 1.11 ^a^	−2.12 ± 0.22 ^b^	28.92 ± 1.25 ^a^
SHA	69.67 ± 0.27 ^a^	−1.57 ± 0.10 ^b^	24.50 ± 0.36 ^bc^	69.69 ± 1.53 ^a^	−0.98 ± 0.04 ^b^	29.12 ± 1.10 ^a^
SHA+vital gluten	72.09 ± 0.73 ^a^	−1.58 ± 0.15 ^b^	24.47 ± 0.23 ^bc^	73.29 ± 1.88 ^a^	−1.43 ± 0.06 ^b^	27.84 ± 1.90 ^a^
rSHA	48.19 ± 1.03 ^b^	3.80 ± 0.13 ^a^	22.36 ± 1.19 ^d^	44.17 ± 1.94 ^b^	7.21 ± 0.33 ^a^	29.54 ± 1.51 ^a^
rSHA+vital gluten	50.22 ± 0.89 ^b^	3.92 ± 0.33 ^a^	23.56 ± 0.69 ^cd^	48.39 ± 1.74 ^b^	8.76 ± 0.83 ^a^	30.94 ± 1.91 ^a^

S: Svevo flour, SHA: Svevo-HA flour, rSHA: recombined flour from heat-treated Svevo-HA starch and Svevo-HA gluten. Means with different letters within each column are significantly different (*p* < 0.05).

**Table 2:** Textural properties of the bazlama samples.

Flours Used in Bazlama	Hardness (N)	Springiness	Cohesiveness	Gumminess	Chewiness	Resilience
S	20.56 ± 0.90 ^e^	0.98 ± 0.01 ^a^	0.88 ± 0.01 ^ab^	18.73 ± 0.35 ^c^	18.39 ± 0.36 ^b^	0.59 ± 0.01 ^ab^
S+vital gluten	17.29 ± 0.69 ^f^	0.98 ± 0.01 ^a^	0.91 ± 0.05 ^a^	14.71 ± 0.96 ^c^	14.30 ± 0.87 ^c^	0.62 ± 0.01 ^a^
SHA	31.44 ± 1.02 ^c^	0.97 ± 0.01 ^a^	0.82 ± 0.01 ^b^	22.48 ± 1.08 ^b^	19.05 ± 0.48 ^b^	0.55 ± 0.03 ^b^
SHA+vital gluten	26.38 ± 1.90 ^d^	0.97 ± 0.01 ^a^	0.84 ± 0.04 ^ab^	21.50 ± 0.89 ^b^	18.45 ± 0.34 ^b^	0.56 ± 0.02 ^b^
rSHA	49.18 ± 0.80 ^a^	0.85 ± 0.07 ^b^	0.77 ± 0.06 ^c^	25.22 ± 0.77 ^a^	22.48 ± 0.31 ^a^	0.43 ± 0.04 ^c^
rSHA+vital gluten	43.86 ± 1.29 ^b^	0.83 ± 0.02 ^b^	0.85 ± 0.01 ^ab^	24.72 ± 0.80 ^a^	21.99 ± 0.75 ^a^	0.47 ± 0.01 ^c^

S: Svevo flour, SHA: Svevo-HA flour, rSHA: recombined flour from heat-treated Svevo-HA starch and Svevo-HA gluten. Means with different letters within each column are significantly different (*p* < 0.05).

**Table 3:** Resistant starch contents, HI, and in vitro GI values of the bazlama samples.

Flours Use	Resistant Starch (% dw)	HI	GI
S	0.96 ± 0.02 ^e^	62.56 ± 0.61 ^ab^	74.06 ± 0.33 ^ab^
S+vital gluten	0.95 ± 0.01 ^e^	63.91 ± 0.46 ^a^	74.80 ± 0.25 ^a^
SHA	2.38 ± 0.07 ^c^	61.22 ± 1.10 ^b^	73.32 ± 0.61 ^b^
SHA+vital gluten	2.08 ± 0.06 ^d^	62.17 ± 0.27 ^b^	73.84 ± 0.15 ^b^
rSHA	10.30 ± 0.07 ^a^	25.63 ± 0.33 ^c^	53.78 ± 0.18 ^c^
rSHA+vital gluten	9.12 ± 0.13 ^b^	26.03 ± 0.35 ^c^	54.00 ± 0.19 ^c^

S: Svevo flour, SHA: Svevo-HA flour, rSHA: recombined flour from heat-treated Svevo-HA starch and Svevo-HA gluten. Means with different letters within each column are significantly different (*p* < 0.05).

**Table 4:** Phenolic content and antioxidant capacities of the bazlama samples.

	Flours Used in Bazlama	Phenolic Contents	ABTS	DPPH	FRAP
Free	S	211.93 ± 0.85 ^d^	34.30 ± 0.45 ^d^	26.18 ± 1.51 ^d^	6.73 ± 0.81 ^c^
	S+vital gluten	218.85 ± 1.28 ^c^	36.85 ± 0.45 ^d^	37.53 ± 0.50 ^c^	11.86 ± 1.61 ^c^
	SHA	219.50 ± 1.65 ^c^	42.28 ± 1.09 ^c^	38.70 ± 0.97 ^c^	20.29 ± 1.56 ^b^
	SHA+vital gluten	232.24 ± 0.42 ^b^	47.90 ± 0.44 ^b^	41.21 ± 1.96 ^c^	24.98 ± 1.58 ^b^
	rSHA	233.07 ± 1.20 ^b^	50.50 ± 0.63 ^b^	46.76 ± 0.94 ^b^	78.37 ± 3.02 ^a^
	rSHA+vital gluten	240.44 ± 0.41 ^a^	63.20 ± 1.08 ^a^	58.64 ± 0.95 ^a^	83.67 ± 3.09 ^a^
Bound	S	300.29 ± 0.64 ^d^	42.41 ± 0.90 ^e^	34.62 ± 2.01 ^e^	51.80 ± 0.97 ^d^
	S+vital gluten	307.96 ± 1.28 ^c^	49.12 ± 1.35 ^d^	41.72 ± 2.01 ^de^	53.17 ± 0.97 ^d^
	SHA	309.16 ± 2.47 ^c^	51.51 ± 0.87 ^d^	47.21 ± 1.94 ^d^	77.22 ± 0.94 ^c^
	SHA+vital gluten	314.36 ± 1.25 ^bc^	68.38 ± 0.79 ^c^	58.14 ± 0.98 ^c^	83.77 ± 1.42 ^b^
	rSHA	319.16 ± 2.99 ^b^	79.77 ± 0.85 ^b^	66.95 ± 1.88 ^b^	87.89 ± 0.45 ^a^
	rSHA+vital gluten	326.97 ± 0.61 ^a^	102.58 ± 1.30 ^a^	81.98 ± 1.92 ^a^	91.07 ± 0.62 ^a^
Total *	S	512.22 ± 1.49 ^d^	76.71 ± 0.45 ^f^	60.81 ± 0.50 ^e^	58.53 ± 1.77 ^d^
	S+vital gluten	526.82 ± 0.10 ^c^	85.97 ± 0.90 ^e^	79.25 ± 1.51 ^d^	65.03 ± 0.65 ^d^
	SHA	528.66 ± 0.82 ^c^	93.79 ± 0.22 ^d^	85.90 ± 2.91 ^d^	97.52 ± 2.50 ^c^
	SHA+vital gluten	546.60 ± 0.83 ^b^	116.29 ± 1.24 ^c^	99.35 ± 0.98 ^c^	108.75 ± 0.16 ^b^
	rSHA	552.23 ± 5.38 ^b^	130.26 ± 0.21 ^b^	113.72 ± 2.82 ^b^	166.26 ± 3.47 ^a^
	rSHA+vital gluten	567.40 ± 0.20 ^a^	165.78 ± 2.38 ^a^	140.62 ± 2.88 ^a^	174.74 ± 3.70 ^a^

S: Svevo flour, SHA: Svevo-HA flour, rSHA: recombined flour from heat-treated Svevo-HA starch and Svevo-HA gluten. Means with different letters within each column are significantly different (*p* < 0.05). The statistical analysis was performed separately within each group, and values followed by different letters in the same column for each group (free, bound, and total) are significantly different (*p* < 0.05). Phenolic contents are expressed as mg gallic acid equivalent (GAE)/100 g dry weight (dw). ABTS: 2,2′-azino-bis(3-ethyl-benzothiazoline-6-sulphonic acid); DPPH: 2,2-diphenyl-1-picrylhydrazyl radical scavenging activity; FRAP: Ferric reducing antioxidant power. * The sum of free and bound antioxidant capacities expressed as mg Trolox equivalent (TE)/100 g dw.

## Data Availability

The original contributions presented in this study are included in the article. Further inquiries can be directed to the corresponding authors.

## References

[1] Jones J.M., Peña R.J., Korczak R., Braun H.J. (2015). Carbohydrates, Grains, and Wheat in Nutrition and Health: An Overview Part I. Role of Carbohydrates in Health. Cereal Foods World.

[2] Bird A.R., Regina A. (2018). High Amylose Wheat: A Platform for Delivering Human Health Benefits. J. Cereal Sci..

[3] Li H., Dhital S., Slade A.J., Yu W., Gilbert R.G., Gidley M.J. (2019). Altering Starch Branching Enzymes in Wheat Generates High-Amylose Starch with Novel Molecular Structure and Functional Properties. Food Hydrocoll..

[4] Topping D.L., Clifton P.M. (2001). Short-Chain Fatty Acids and Human Colonic Function: Roles of Resistant Starch and Nonstarch Polysaccharides. Physiol. Rev..

[5] Jiang H., Campbell M., Blanco M., Jane J.-L. (2010). Characterization of Maize Amylose-Extender (Ae) Mutant Starches: Part II. Structures and Properties of Starch Residues Remaining after Enzymatic Hydrolysis at Boiling-Water Temperature. Carbohydr. Polym..

[6] Jiang H., Jane J., Shi Y., Maningat C.C. (2013). Type 2 Resistant Starch in High-amylose Maize Starch and Its Development. Resistant Starch.

[7] Li C., Dhital S., Gidley M.J. (2022). High-Amylose Wheat Bread with Reduced in Vitro Digestion Rate and Enhanced Resistant Starch Content. Food Hydrocoll..

[8] Belleggia L., Foligni R., Ferrocino I., Biolcati F., Mozzon M., Aquilanti L., Osimani A., Harasym J. (2023). Morphotextural, Microbiological, and Volatile Characterization of Flatbread Containing Cricket (*Acheta domesticus*) Powder and Buckwheat (*Fagopyrum esculentum*) Flour. Eur. Food Res. Technol..

[9] Beyaz S., Cetiner B., Ozkan K., Sagdic O., Sestili F., Koksel H. (2025). A Functional Flatbread (Bazlama): High in Beta-Glucan and Plant-Based Protein Content. Foods.

[10] Koksel H., Tekin-Cakmak Z.H., Oruc S., Kilic G., Ozkan K., Cetiner B., Sagdic O., Sestili F., Jilal A. (2024). A New Functional Wheat Flour Flatbread (Bazlama) Enriched with High-β-Glucan Hull-Less Barley Flour. Foods.

[11] Ertaş N. (2016). The Effect of Microwave, Autoclave and Hot Air Oven Stabilized Wheat Bran Substitution on Nutritional and Sensorial Propoerties of Flat Breads. J. Food Health Sci..

[12] Yamada Y., Hosoya S., Nishimura S., Tanaka T., Kajimoto Y., Nishimura A., Kajimoto O. (2005). Effect of Bread Containing Resistant Starch on Postprandial Blood Glucose Levels in Humans. Biosci. Biotechnol. Biochem..

[13] American Association of Cereal Chemists (2010). Approved Methods of the American Association of Cereal Chemists.

[14] Koksel H., Tekin-Cakmak Z.H., Ozkan K., Pekacar Z., Oruc S., Kahraman K., Ozer C., Sagdic O., Sestili F. (2024). A Novel High-Amylose Wheat-Based Functional Cereal Soup (Tarhana) with Low Glycemic Index and High Resistant Starch. J. Cereal Sci..

[15] Kahraman K., Aktas-Akyildiz E., Ozturk S., Koksel H. (2019). Effect of Different Resistant Starch Sources and Wheat Bran on Dietary Fibre Content and In Vitro Glycaemic Index Values of Cookies. J. Cereal Sci..

[16] Tekin-Cakmak H.Z., Ozer C., Ozkan K., Yildirim H., Sestili F., Jilal A., Sagdic O., Ozgolet M., Koksel H. (2024). High-Beta-Glucan and Low-Glycemic Index Functional Bulgur Produced from High-Beta-Glucan Barley. J. Funct. Foods.

[17] Re R., Pellegrini N., Proteggente A., Pannala A., Yang M., Rice-Evans C. (1999). Antioxidant Activity Applying an Improved ABTS Radical Cation Decolorization Assay. Free Radic. Biol. Med..

[18] Singh R.P., Chidambara Murthy K.N., Jayaprakasha G.K. (2002). Studies on the Antioxidant Activity of Pomegranate (*Punica granatum*) Peel and Seed Extracts Using In Vitro Models. J. Agric. Food Chem..

[19] Benzie I.F.F., Strain J.J. (1996). The Ferric Reducing Ability of Plasma (FRAP) as a Measure of “Antioxidant Power”: The FRAP Assay. Anal. Biochem..

[20] Bhoir S.A., Sharma D., Jamdar S. (2025). Millets, Pulses, and Oil Seeds-based Flatbread Premix: A Protein-rich Functional Food for Healthier Dietary Habits and Prevention of Lifestyle Disorders. J. Food Sci..

[21] Gomathi G.K., Parameshwari S. (2022). Evaluation of Buckwheat Flour Addition on the Sensory, Nutritional and Materialistic Properties Analysis of Indian Flat Bread. Mater. Today Proc..

[22] Şanal B.N., Şahin N., Sayaslan A. (2025). Development of Gluten-Free Bazlama Bread Enriched with Chickpea Flour. J. Culin. Sci. Technol..

[23] Andrzej K.M., Małgorzata M., Sabina K., Horbańczuk O.K., Rodak E. (2020). Application of Rich in β-Glucan Flours and Preparations in Bread Baked from Frozen Dough. Food Sci. Technol. Int..

[24] Cacak-Pietrzak G., Sujka K., Księżak J., Bojarszczuk J., Ziarno M., Studnicki M., Krajewska A., Dziki D. (2024). Assessment of Physicochemical Properties and Quality of the Breads Made from Organically Grown Wheat and Legumes. Foods.

[25] de la Hera E., Rosell C.M., Gomez M. (2014). Effect of Water Content and Flour Particle Size on Gluten-Free Bread Quality and Digestibility. Food Chem..

[26] Kumar A., Sahoo U., Baisakha B., Okpani O.A., Ngangkham U., Parameswaran C., Basak N., Kumar G., Sharma S.G. (2018). Resistant Starch Could Be Decisive in Determining the Glycemic Index of Rice Cultivars. J. Cereal Sci..

[27] Matos Segura M.E., Rosell C.M. (2011). Chemical Composition and Starch Digestibility of Different Gluten-Free Breads. Plant Foods Hum. Nutr..

[28] Ge X., Jing L., Zhao K., Su C., Zhang B., Zhang Q., Han L., Yu X., Li W. (2021). The Phenolic Compounds Profile, Quantitative Analysis and Antioxidant Activity of Four Naked Barley Grains with Different Color. Food Chem..

[29] Mazhar S.H., Waseem M., Ahmad Z., Javed M.R., Manzoor M.F., Khan M.A., Mugabi R., Alsulami T., Nayik G.A. (2024). Influence of Microwave Processing on Nutritional, Anti-Nutritional, Antioxidant and Sensory Characteristics of Kachnar Powder and Supplemented Flatbreads. Food Chem. X.

